# Supplementary material for: Widespread redundancy in -omics profiles of cancer mutation states
Source: Genome Biol. 2022 Jun 27;23:137. doi: 10.1186/s13059-022-02705-y (PMC9238138; doi:10.1186/s13059-022-02705-y)
Supplement: Supplementary file 1 — Additional file 1. All supplementary figures and supplementary information. [file 13059_2022_2705_MOESM1_ESM.pdf]

Supplementary material for “Widespread redundancy in -omics profiles of cancer mutation states”

A version of the main paper figures using the area under the receiver-operator curve (AUROC) metric rather than AUPR is available at `https://doi.org/10.6084/m9.figshare.14919729`.

In a previous version of this paper, we ran our analysis only for the genes in the Vogelstein et al. 2013 gene set. While there were some gene-to-gene differences in this set, we did not observe large differences between methylation and gene expression performances overall. Scaling up the gene set by combining cancer gene sets from the literature as described in the methods/results sections affected the study results somewhat, as mutations in the added genes tend to be better predicted using gene expression than other data types. During the revision, we explored the difference between the genes in this gene set and the genes in the “merged” cancer-related gene set but not in the Vogelstein genes. GO analysis results for the Vogelstein genes are available at `https://doi.org/10.6084/m9.figshare.19565890`, and results for the non-Vogelstein genes are available at `https://doi.org/10.6084/m9.figshare.19565887`. We noticed that the non-Vogelstein genes tend to be enriched for terms relating to transcription factors and transcriptional regulation.

As a data resource, coefficients and hyperparameter choices for final models fit using all data types are available on Figshare: coefficients are available at `https://doi.org/10.6084/m9.figshare.19576012` and hyperparameters are at `https://doi.org/10.6084/m9.figshare.19576048`. Columns in the coefficients dataset correspond to target genes (gene symbols), and rows correspond either to PCA components (for 27K and 450K methylation), -omics features (for all other data types), or covariates (cancer type indicator variables or log(mutation burden)). An ‘NA’ value in a cell indicates that feature was not used in the model for the corresponding gene (for an -omics feature this could mean it was not in the top 8000 features by MAD, for a cancer type feature this means that cancer type was not included in the training set based on our mutation filters). A 0 value in a cell indicates that feature was included in model training, but it was not selected by the elastic net feature selection algorithm. Columns in the hyperparameters dataset correspond to hyperparameters (alpha and l1_ratio for elastic net logistic regression) and rows correspond to target genes. For the methylation data types, PCA results (score and loading matrices) corresponding to the coefficients data are also available at `https://doi.org/10.6084/m9.figshare.19908034`. These contain the top 5,000 principal components for each data type, which were used in the classifiers evaluated in the main paper.

Regarding the hyperparameters for the final models, recall that for the main figures in the paper, we evaluate each of our models using 2 replicates of 4-fold cross-validation. For each of these folds (train/test splits), we further split the training set into train and validation sets to select hyperparameters, independently for each fold, and evaluate the models on the test set to get the results in the paper. Because we are evaluating performance over multiple folds, it is not perfectly straightforward to get a single set of regression coefficients, since we have a (potentially different) set of coefficients for each cross-validation fold. In order to synthesize these results into a single model for each gene in each data type, we selected one of the 8 sets of hyperparameters (from the 8 best models, 1 per CV fold) at random, with probability proportional to performance (AUPR) on the validation set used to select the hyperparameters, described above (so test set performance is not used here). We then used the selected hyperparameters to train a single model on the entire dataset.

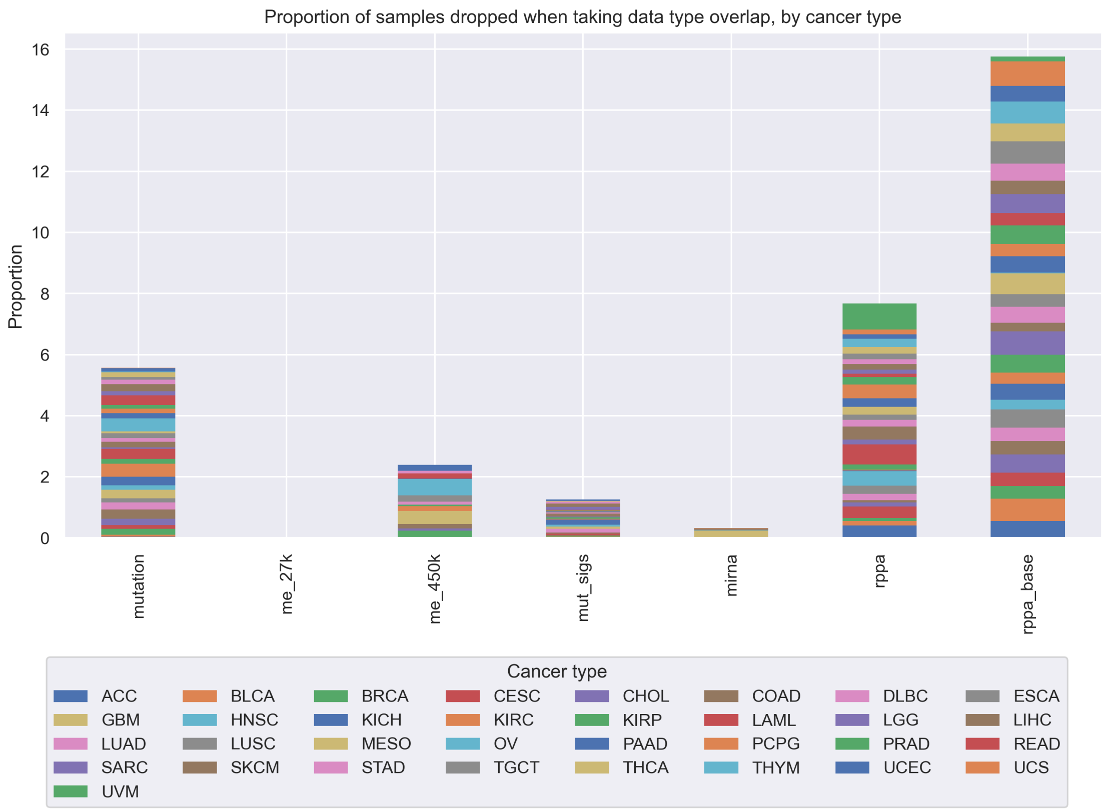

Figure S1: Proportion of samples from each TCGA cancer type that are "dropped" as more data types are added to our analyses. We started with gene expression data, and for each added data type, we took the intersection of samples that were profiled for that data type and the previous data types, dropping all samples that were missing 1 or more data types. Overall, at each step, the proportions of "dropped" samples appear to be fairly evenly spread between cancer types, showing that in general we are not disproportionately losing one or several cancer types as more data modalities are added to our analyses.

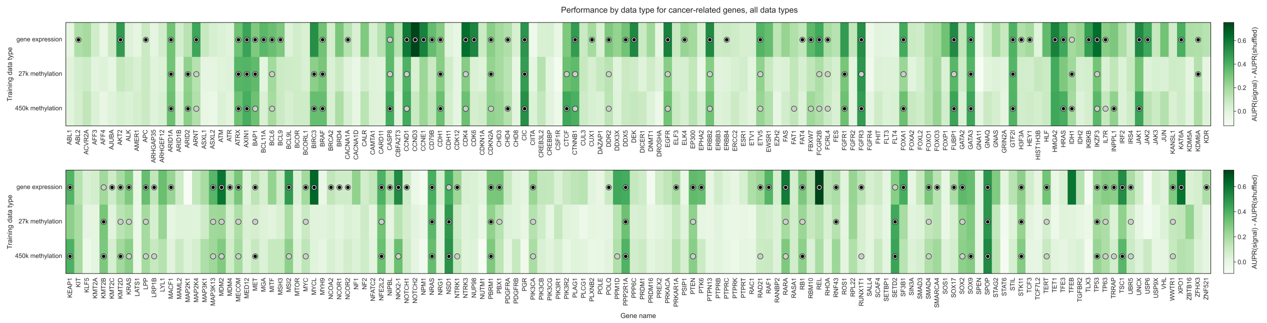

Figure S2: Heatmap displaying predictive performance for mutations in each of the 272 genes from the cancer-related gene set, across gene expression and the two DNA methylation arrays. Each cell quantifies performance for a target gene, using predictive features derived from a particular data type. Grey shaded dots indicate that the given data type provides significantly better predictions than the permuted baseline for the given gene; black inner dots indicate the same and additionally that the given data type provides statistically equivalent performance to the data type with the best average performance (determined by pairwise t-tests across data types with FDR correction).

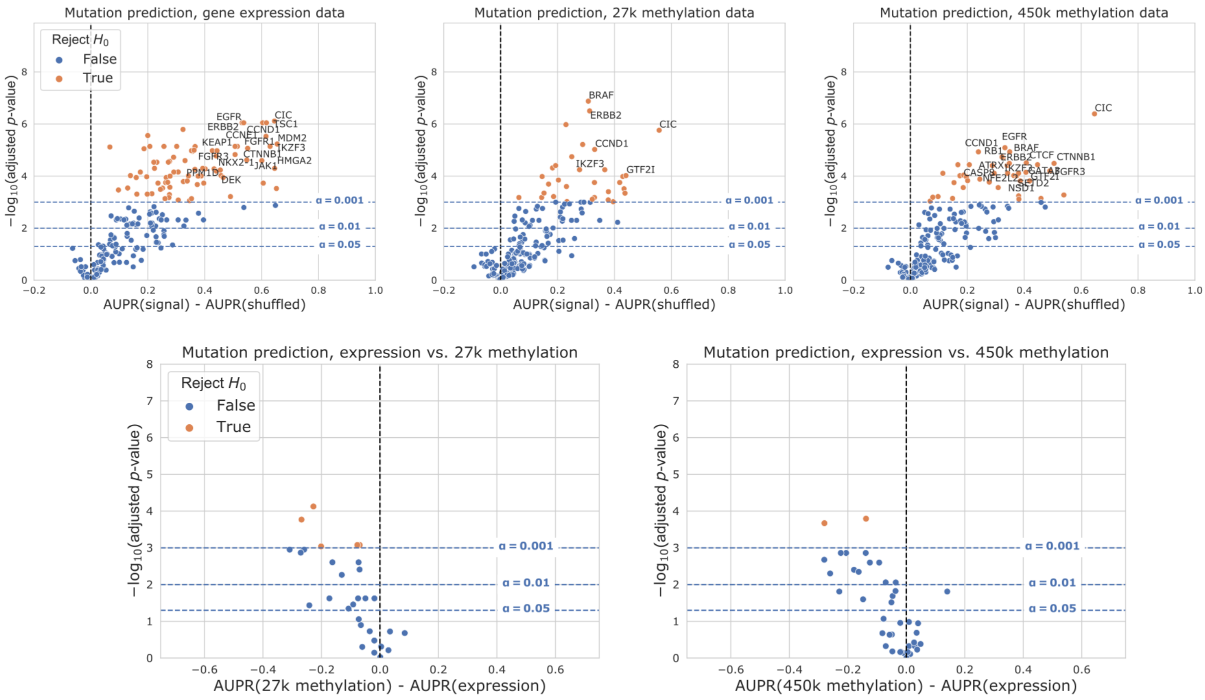

Figure S3: Volcano-like plots showing predictive performance for each gene in the cancer-related gene set for expression and DNA methylation, on the sample set used for the "all data types" experiments. The first row shows performance relative to the permuted baseline, and the second row shows direct comparisons between data types for genes that outperformed the permuted baseline only for both data types. The x-axis shows the difference in mean AUPR compared with a baseline model trained on permuted labels, and the y-axis shows p-values for a paired t-test comparing cross-validated AUPR values within folds.

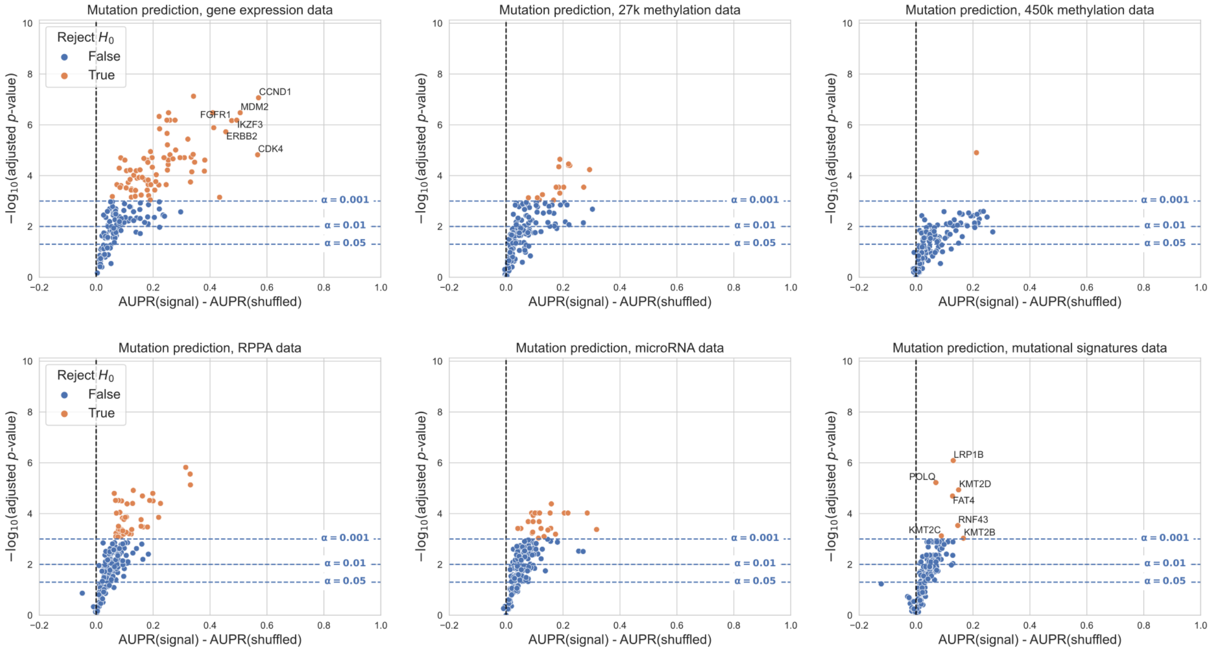

Figure S4: Volcano-like plots showing predictive performance for each gene in the cancer-related gene set for all data types, relative to the permuted baseline model, when genes are filtered based on the entire dataset rather than by cancer type. For this filtering approach, we included/excluded entire genes rather than individual cancer types: specifically, we trained a classifier for each gene where all cancer types combined had at least 5% mutated samples and at least 100 total mutated samples, resulting in 182 total classifiers. The x-axis shows the difference in mean AUPR compared with a baseline model trained on permuted labels, and the y-axis shows p-values for a paired t-test comparing cross-validated AUPR values within folds. Counts of genes making the significance threshold of 0.001: gene expression 81/182 (44.5%), 27K methylation 16/182 (8.8%), 450K methylation 1/182 (0.6%), RPPA 41/182 (22.5%), microRNA 25/182 (13.7%), mutational signatures 7/182 (3.9%).

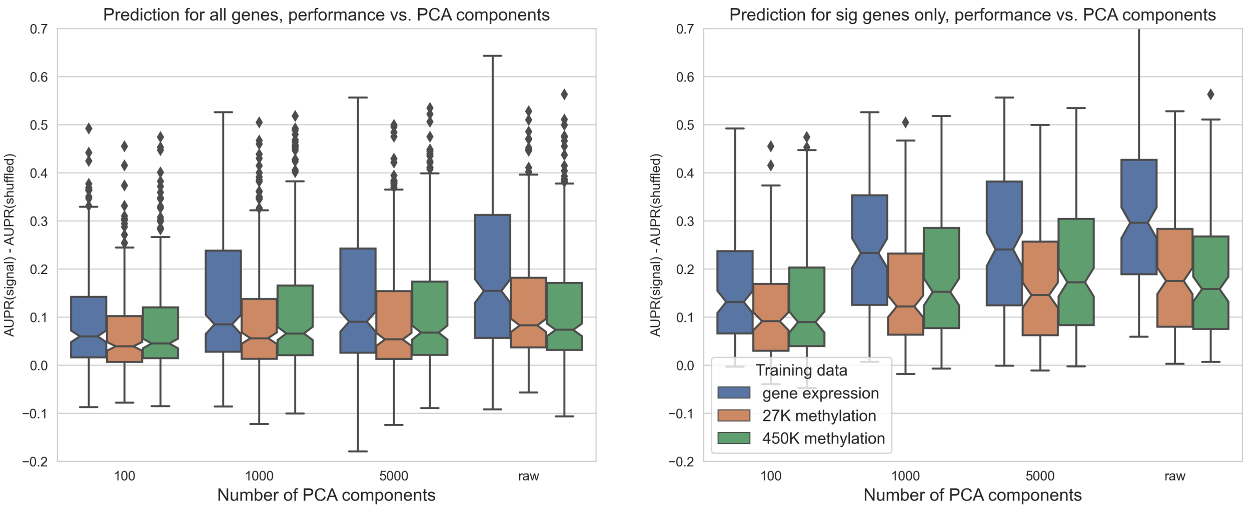

Figure S5: Predictive performance for genes in the cancer-related gene set, using each of the three data types as predictors. The x-axis shows the number of PCA components used as features, “raw” = no PCA compression.

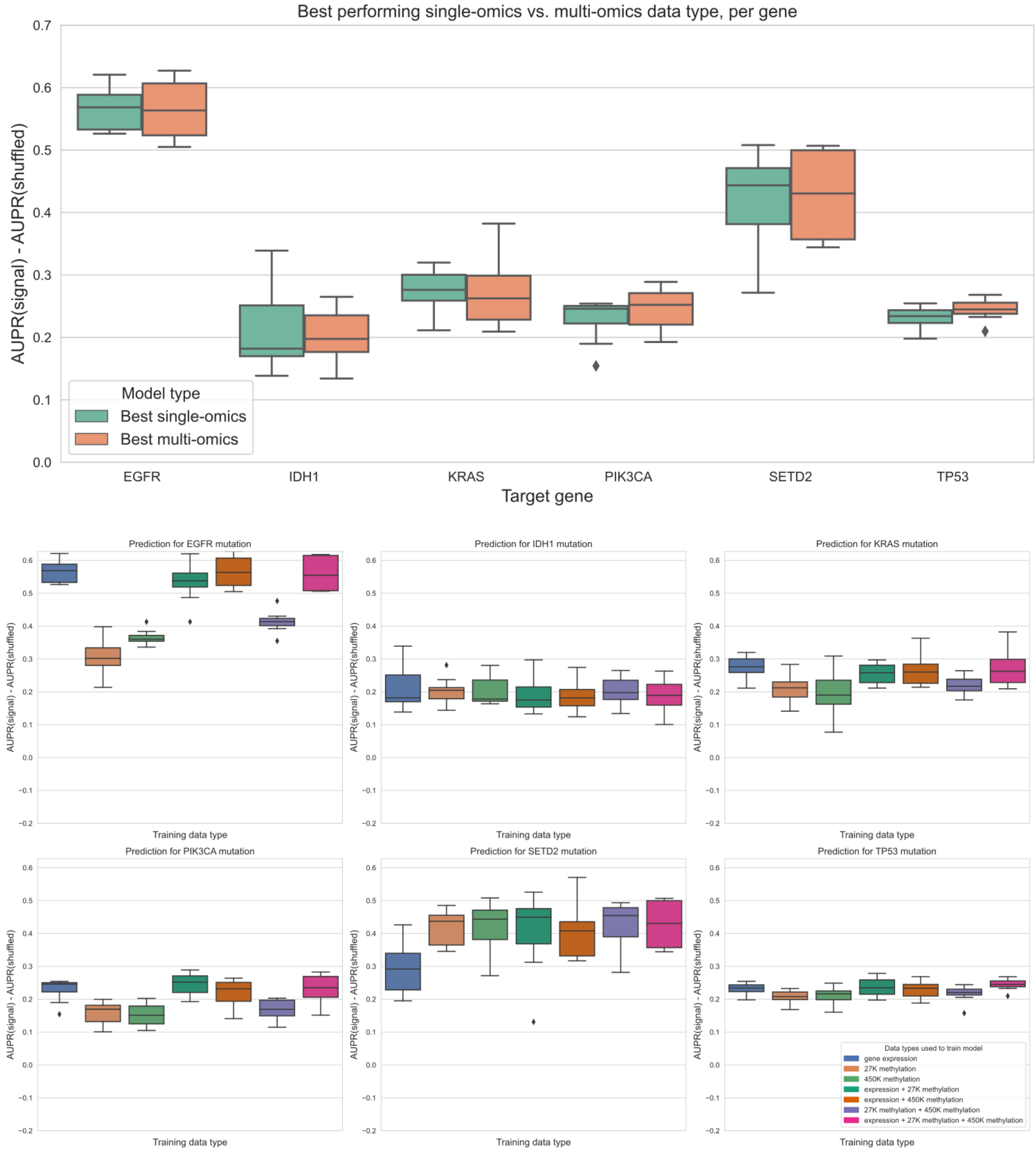

Figure S6: Top plot: comparing the best-performing model (i.e. highest mean AUPR relative to permuted baseline) trained on a single data type against the best "multi-omics" model for each target gene, using raw (not PCA compressed) features. For feature parity between data types, the top 20,000 features by mean absolute deviation were used for each data type. The difference between single-omics and multi-omics performance for TP53 was statistically significant (p=0.0078), but other differences between single-omics and multi-omics models were not statistically significant using paired-sample Wilcoxon tests across cross-validation folds, for a threshold of 0.05. Bottom plots: classifier performance, relative to baseline with permuted labels, for individual genes. Each panel shows performance for one of the six target genes; box plots show performance distribution over 8 evaluation sets (4 cross-validation folds x 2 replicates).

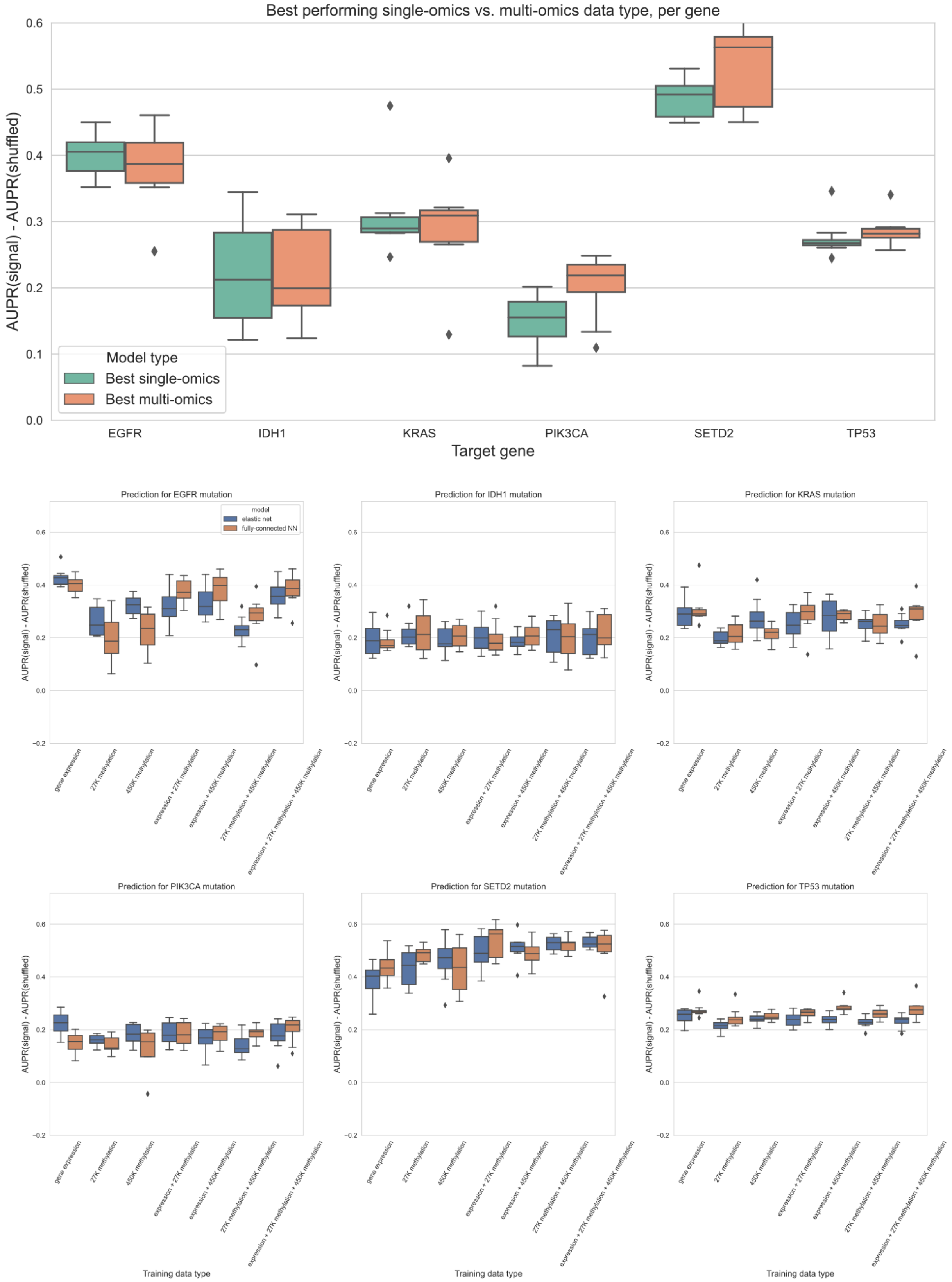

Figure S7: Top plot: comparing the best-performing model (i.e. highest mean AUPR relative to permuted baseline) trained on a single data type against the best “multi-omics” model for each target gene, using a 3-layer fully-connected neural network. The top 5,000 principal components were used as predictive features for each data type. The difference between single-omics and multi-omics performance for PIK3CA ($p = 0.0156$, in favor of multi-omics) and TP53 ($p = 0.0391$, in favor of single-omics) were statistically significant, but other differences between single-omics and multi-omics models were not statistically significant using paired-sample Wilcoxon tests across cross-validation folds, for a threshold of 0.05. Bottom plots: comparison of classifier performance between elastic net and fully-connected NN, relative to baseline with permuted labels, for individual genes. Each panel shows performance for one of the six target genes; box plots show performance distribution over 8 evaluation sets (4 cross-validation folds x 2 replicates).
