# Supplementary material for: Widespread redundancy in -omics profiles of cancer mutation states
Source: Genome Biol. 2022 Jun 27;23:137. doi: 10.1186/s13059-022-02705-y (PMC9238138; doi:10.1186/s13059-022-02705-y)
Supplement: Supplementary file 3 — Additional file 3. Review history. [file 13059_2022_2705_MOESM3_ESM.docx]

Review History

**First round of review**

**Reviewer 1**

**Were you able to assess all statistics in the manuscript, including the appropriateness of statistical tests used?**

Yes, and I have assessed the statistics in my report.

**Comments to author:**

In this work, authors trained logistic regression classifiers to predict a mutational event in a target gene using distinct -omic readout. Overall, their RNA-seq and DNA methylation predictors performed significantly better than other readouts. Furthermore, they observed that their trained predictor based on multi-omics data showed little or no improvement in predictive performance compared to the best predictor using single -omic readout. Although the current study is interesting, I have some significant concerns about the manuscript in the current form.

1. Authors are using their RNA-seq model to predict the mutational status of a target gene. One could also identify somatic mutations using RNA-seq data. How consistent is their prediction concerning the mutational status of target genes based on mutation calling using RNA-seq?

2. Can authors show that their model performs well for genes belonging to DNA-repair or cancer-related pathways that do not overlap with Vogelstein's driver gene list?

3. For gene-level prediction analysis described in Fig4, is there any biological interpretation behind differences in prediction accuracy for different genes?

4. How do technical factors, including differences in data quality for distinct -omics readout, influence the prediction performance comparison as reported in Fig5.

5. Authors indicate that they are using a fairly simplistic model for multi-omics integration to predict the mutational status of a target gene. Can authors use a more sophisticated model including a deep neural network to perform the same comparison?

**Reviewer 2**

**Were you able to assess all statistics in the manuscript, including the appropriateness of statistical tests used?**

Yes, and I have assessed the statistics in my report.

**Comments to author:**

In this paper, the authors investigate the utility of different -omics datasets in predicting cancer driver gene mutation status as well as patient survival. They find associations for gene expression and methylation. Overall, the manuscript is well written and the authors present a careful statistical analysis. In particular, they account for spurious signals in the AUPR by computing the difference in AUPR relative to that of shuffled data. Also, multiple testing is properly accounted for within individual panels. The observed associations are interesting.

1. The observed predictability of driver gene mutation status by expression data can have many different origins, including nonsense-mediated decay, overexpression of certain oncogenes and differential gene regulation if the mutated genes include transcription factors. In this regard, there are two important additional tests that are currently missing:

i. Investigate whether the correlation between driver gene mutation status and expression is driven by the expression of the mutated genes themselves (i.e. whether this is the most predictive feature in the expression vector). To this end, one should repeat the analysis excluding the gene whose mutation status is being tested.

ii. Investigate whether the prediction is driven by the expression landscape of the tissue of origin. To this end, the analysis should be repeated with expression levels from healthy cells of the tissue of origin of the tumor.

2. Along similar lines, what are the most important features (i.e. largest regression coefficients)?

3. p.7, l. 6: The current COSMIC cancer gene census contains 578 tier 1 genes that have been associated with cancer (https://cancer.sanger.ac.uk/cosmic/census?tier=1). Why do the authors restrict to the list given in Vogelstein et al. from 2013, which only contains 127 genes and is further reduced to only 85? In particular, the extended list will contain a lot more DNA repair genes.

4. p. 13, l. 43: Why is it that in this paragraph 100 Vogelstein genes were analyzed instead of 85 or less (p. 7, l.7)?

5. p. 7, l. -1: "we included only cancer types with at least 15 mutated samples and at least 5% mutated samples, which we refer to here as "valid" cancer types." Why was there an exclusion criterion for entire cancer types (as opposed to just filtering based on sample properties)?

6. p. 12, l. 7: It is stated that the set of 85 random genes contains eight of the Vogelstein gene set. This implies that the total number of genes that were used (and from which the random genes were drawn) is only on the order of 1,000 genes. What happened with the remaining ~18,000 human protein-coding genes? Why were they excluded?

7. Figure 7: How come the models that include more features sometimes perform worse than nested models with fewer features? In particular, gene expression on its own seems to significantly outperform the models that include expression plus additional variables. Is this due to the choice of hyperparameters or due to something else?

8. p. 9, l. 40: Here, it is mentioned that individual cancer types were analyzed as well. The only figure that shows results for individual cancer types is Suppl. Fig. 12. However, this figure is not mentioned in the main text. Also, it only shows five cancer types and only the predictions for survival. The analysis of the other cancer types is missing, and so is the analysis of the prediction of cancer-type-specific driver mutations.

9. Suppl. Fig. 12: It is not quite clear to me what is the difference in the prediction of survival by using, say, gene expression in the upper panel vs in the lower panel. Yet, in the upper panel the -omics datasets can predict survival better than the baseline covariates (patient age and mutation count), while in the lower panel they are performing worse. Are these not always single-observable regressions?

10. I think the most interesting application of the approach would be to predict mutation status of all ~19,000 human protein-coding genes to see if the association with expression level can identify novel cancer driver genes. But it is appreciated that the multiple testing burden would probably overwhelm any signal (including the one on the known cancer driver genes).

11. p. 18, l. 5: 36 --> 34

12. p. 18, l. 12: There are no red boxes in Figure 6.

**Authors Response**

**Point-by-point responses to the reviewers’ comments:**

*We thank both reviewers for the helpful and constructive feedback. We believe that we were able to address all of the issues raised, and considering the reviewers’ feedback has considerably improved the study from our perspective. To summarize the major changes:*

- *We have revised Figures 2-6 to use a larger and more representative set of cancer-related genes, which combines multiple sets of cancer genes from the literature including, but not limited to, the Vogelstein et al. dataset from the original study, and revised the corresponding text throughout the manuscript. To further explore the differences between gene sets, we performed a GO enrichment analysis, and we have provided the results as a data resource in the supplement of the revised manuscript.*
- *We have compiled coefficients and hyperparameters for gene expression-based classifiers trained on all of the genes in Figure 5, and we have added them to the supplement in the revised manuscript as a data resource.*
- *For the multi-omics classification experiment in Figure 7, we trained a fully-connected neural network and compared it with the elastic net logistic regression results shown in the Results section. We have edited the text in the Results section and added Supp. Figure 14 to summarize the results.*
- *We explored an alternate method for filtering genes to train classifiers on, by gene across all cancer types rather than by cancer type, and we have explained and summarized the results in Supp. Figure 11.*
- *We added text to the Discussion section noting mutation calling directly from RNA-seq reads as a possibility in certain settings.*
- *We have removed the previous Supp. Figure 12 from the revised version of the manuscript, since it was an exploratory/diagnostic analysis that was not referenced in the main text.*

*Our responses to individual points can be found below:*

Reviewer #1:

In this work, authors trained logistic regression classifiers to predict a mutational event in a target gene using distinct -omic readout. Overall, their RNA-seq and DNA methylation predictors performed significantly better than other readouts. Furthermore, they observed that their trained predictor based on multi-omics data showed little or no improvement in predictive performance compared to the best predictor using single -omic readout. Although the current study is interesting, I have some significant concerns about the manuscript in the current form.

1. Authors are using their RNA-seq model to predict the mutational status of a target gene. One could also identify somatic mutations using RNA-seq data. How consistent is their prediction concerning the mutational status of target genes based on mutation calling using RNA-seq?

*Reply: We appreciate the reviewer’s thoughtful suggestion. While we think the question of whether or not our pipeline for training mutation status classifiers would work with RNA-seq data alone is interesting, we also think it may help to slightly clarify the main goals of our study.*

*We are primarily concerned with improving our understanding of the functional effects of cancer mutations (using RNA-seq, DNA methylation, proteomics data, etc as readouts of cellular function in cancer, and understanding their relative benefits/drawbacks in this context), as opposed to identifying new cancer mutations/driver genes or training classifiers that will be used directly for genotyping. For this application, we believe that mutation calling from DNA sequencing data is the best gold standard we have access to (i.e. as a proxy for cell state, or a source of labels for our classifiers), and mutations called from RNA sequencing data would be a noisier readout of cell state. Establishing that our predictions are consistent when mutations are called using RNA-seq data, then, would not necessarily tell us anything new about cell function in cancer.*

*Additionally, in the process of revising our manuscript, we briefly discussed mutation calling from RNA-seq data with a collaborator who works in this area. In our collaborator’s opinion, calling cancer somatic mutations accurately from RNA-seq data is an open question, and there does not seem to be a standard pipeline for doing this well. Given these paired considerations, we have added a section in the discussion (toward the end of the second paragraph on Page 19: beginning with “Although the mutation…”) noting calling directly from RNA-seq reads as an option in appropriate settings.*

2. Can authors show that their model performs well for genes belonging to DNA-repair or cancer-related pathways that do not overlap with Vogelstein's driver gene list?

*Reply: We thank the reviewer for calling our attention to the fact that the Vogelstein driver gene list is limited in its scope and functional coverage. Following the related comment from Reviewer #2 suggesting that we consider the COSMIC Cancer Gene Census gene set, which covers a much broader set of cancer-associated genes including DNA repair genes, we constructed a “merged” set of cancer genes consisting of the union of the genes from Vogelstein et al. 2013, COSMIC CGC, and driver genes identified as “pan-cancer” in Bailey et al. 2018 (the paper describing the TCGA effort to identify and characterize driver genes).*

*This resulted in an initial set of 511 total cancer genes, considerably larger than our initial set of 125 genes from the Vogelstein et al. study. We re-ran all of the single-omics analyses from the paper on this larger set of cancer genes, and edited the figures and text in the main paper to reflect the updated conclusions. To summarize:*

- *On the larger set of genes, we generally see a larger performance advantage for gene expression, relative to the other data types, than we did in the initial study on the Vogelstein et al. dataset. For* *many genes (including those in the original Vogelstein et al. dataset) gene expression and DNA methylation still provide similar predictive performance, but in general across the larger gene set gene expression tends to perform best more often.*
- *We performed a GO molecular function and biological process enrichment analysis on the added genes, in order to identify potential biological reasons behind this change in relative performance. In general, we observed that the added genes tend to be enriched for GO terms relating to transcription factor activity, TF binding, and DNA binding, which may explain the predictive ability of RNA-seq for mutations in these genes. A description of the GO analysis, as well as a list of the significantly enriched terms for both Vogelstein and non-Vogelstein genes, can be found in the supplementary information for the revised paper.*

*To answer the original question, asking whether or not our approach generalizes outside of the Vogelstein gene set, in slightly more depth, we visualized the results for the added genes (i.e. genes that are in the “merged” gene set but not in the Vogelstein gene set). Here are the results for gene expression and methylation:*

*Counting the genes that make the significance threshold α=0.001 (after applying a multiple testing correction to the whole gene set before filtering to the added genes), we see that* ***45/142 (31.6%)*** *of “non-Vogelstein” genes make the threshold for gene expression, as compared to* ***9/142 (6.34%)*** *and* ***16/142 (11.3%)*** *of genes for 27K and 450K methylation respectively. Relative to the numbers on the Vogelstein data –* ***34/75 (45.3%), 22/75 (29.3%),*** *and* ***26/75 (34.7%)*** *for expression, 27K, 450K in that order – we can conclude that performance on cancer genes outside the Vogelstein dataset (but in the other cancer datasets we included) is somewhat consistent with the Vogelstein genes for gene expression, but drops off considerably for DNA methylation.*

*This result is not what we initially would have expected! We again thank both reviewers for bringing up the limited scope of the Vogelstein gene set and identifying key alternatives and additions. Our conclusions in this more general version of our analysis, and the recommendations for study design/data readout selection that follow from our results, will be much more robust and generalizable.*

3. For gene-level prediction analysis described in Fig4, is there any biological interpretation behind differences in prediction accuracy for different genes?

*Reply: We agree that the biological interpretation of varying classifier accuracy is an interesting question underlying the results in this study. One of the main takeaways for us, and a result that initially surprised us, was that in many driver genes with a large effect all -omics layers are perturbed strongly, and thus are highly predictive of mutation status in that gene. Consider, for instance, IDH1: we initially included it as an example of a gene that is highly involved in epigenetic regulation (i.e. regulation of both histone marks and DNA methylation patterns), so we assumed that DNA methylation would be highly predictive of mutation status, potentially more so than other data types. DNA methylation data is indeed highly predictive, but in Figure 6 one can see that gene expression, RPPA, and microRNA were also highly predictive. In this case our biological interpretation would be that all of these functional readouts are highly interconnected, so when mutations in a gene such as IDH1 are involved in carcinogenesis and cancer progression in a major way, they tend to affect all layers of cellular function in a detectable way. We hope we were able to emphasize this interpretation in the revised manuscript.*

*Many of the other genes in Figure 4 follow similar trends, in the sense that it would be hard to predict a priori which data type would be most useful for prediction, and for most of them more than one data type is significantly predictive. In general, this is one of the messages we hope to get across with this study, and we hope that the resources we have provided with the revised paper (including the heatmaps in Figure 6 and Supplementary Figure 9, and the model coefficients now provided in the supplementary data) will help researchers to further investigate this question, and apply it to their own experimental design, in a data-driven manner.*

4. How do technical factors, including differences in data quality for distinct -omics readout, influence the prediction performance comparison as reported in Fig5.

*Reply: One of the advantages of using data from TCGA, and a major reason we decided to use it for this study, is that the data processing pipelines used are fairly standard, and they tend to be applied uniformly across data sources/sites. We aren’t aware of any data quality metrics included with the TCGA data specifically, so a comparison of the quality of specific datasets used in this study would be difficult.*

*Addressing the question more generally, it is possible that technical differences in the ability to measure certain data layers, or the technologies chosen by TCGA to measure various aspects of cell state, do influence the comparison. For instance, the 27K and 450K methylation arrays used in this study are useful for cheap, high-throughput profiling of CpG sites, but they could miss informative parts of the “methylome” that a more expensive but more comprehensive technology such as whole-genome bisulfite sequencing would capture. RPPA, the proteomics technology used by TCGA/TCPA, is also scalable to many samples but very limited in its scope/breadth compared to mass spectrometry-based proteomics, although the antibodies used to quantify protein abundance were chosen to cover known cancer-related pathways and protein modifications. In this sense, we are limited by the data made available through TCGA, and we have added a line to the discussion pointing out the possibility that this influences the comparison (last paragraph on the middle of page 20, “It is important to note that the specific technologies…”) However, we think the advantages of large sample size and relatively uniform data processing justify its use.*

5. Authors indicate that they are using a fairly simplistic model for multi-omics integration to predict the mutational status of a target gene. Can authors use a more sophisticated model including a deep neural network to perform the same comparison?

*Reply: We appreciate the suggestion from the reviewer. To explore the use of more sophisticated multi-omics models that consider interactions between features, we trained a fully-connected 3-layer neural network to predict mutation status from either single-omics or multi-omics data. For each data type, we used the top 5,000 principal components as input. We selected hyperparameters for each of the 8 outer cross-validation splits using a single inner train/validation split and a random search over 20 hyperparameter combinations. Implementation and training details, and hyperparameters that we searched over, are described in detail in the revised Methods section (page 9, second paragraph).*

*The results are shown in Supplementary Figure 14 of the revised paper, reproduced below:*

*The top plot shows the best-performing single-omics and multi-omics model for each gene (analogous to Figure 7A), and the bottom plot shows classifier performance for each gene and each data type for the elastic net and NN models (similar to Figure 7B-G). In the top plot, the difference between single-omics and multi-omics performance for PIK3CA (p = 0.0156, in favor of multi-omics) and TP53 (p = 0.0391, in favor of single-omics) were statistically significant after multiple testing correction, but other differences between single-omics and multi-omics models were not statistically significant.*

*In the bottom plot, we observed that the neural network model (orange boxes) sometimes provides an improvement over the elastic net model, particularly for the multi-omics input data (e.g. for EGFR and TP53, and somewhat for PIK3CA). However, the neural network model also sometimes performs worse, especially on the single-omics inputs, and it tends to have higher variability across cross-validation folds (box plots) than the elastic net models. We chose to keep the elastic net results in the main paper because they tend to be more robust across cross-validation folds and require less tuning of hyperparameters, but we included a description in the revised Results section referring to the neural network experiments, as well as the supplementary figure and Methods description mentioned above.*

*In general, the results of the neural network experiments are promising, particularly the improved performance for multi-omics data in some cases, and we think this hints at the value of incorporating interactions between multi-omics features. We would like to emphasize that our goal in this study is not to develop the top-performing method to predict mutation status from multi-omics data. The main message of the multi-omics component of the study, to us, is not that it is not possible to develop a better model, but rather that simple methods do not provide a large performance gain, and that the problem of predicting mutation status from multi-omics data could be a focus for future development of more complex models. In this sense, we think the added experiments do suggest that there may be some nonlinear signal in the data that could be captured more effectively by a more sophisticated model, and we thank the reviewer for the suggestion.*

Reviewer #2:

In this paper, the authors investigate the utility of different -omics datasets in predicting cancer driver gene mutation status as well as patient survival. They find associations for gene expression and methylation. Overall, the manuscript is well written and the authors present a careful statistical analysis. In particular, they account for spurious signals in the AUPR by computing the difference in AUPR relative to that of shuffled data. Also, multiple testing is properly accounted for within individual panels. The observed associations are interesting.

1. The observed predictability of driver gene mutation status by expression data can have many different origins, including nonsense-mediated decay, overexpression of certain oncogenes and differential gene regulation if the mutated genes include transcription factors. In this regard, there are two important additional tests that are currently missing:

i. Investigate whether the correlation between driver gene mutation status and expression is driven by the expression of the mutated genes themselves (i.e. whether this is the most predictive feature in the expression vector). To this end, one should repeat the analysis excluding the gene whose mutation status is being tested.

*Reply: We appreciate the suggestion from the reviewer. To test this, we used gene expression (RNA-seq) data from TCGA to compare classifiers trained on the full gene feature set against classifiers trained on the same set of gene features without the target gene. In other words, the models in the latter experiment* *(referred to as the “drop target” experiment in the plot) have no information about the dosage of the target gene whose mutation status we aim to predict.*

*The following plot shows the difference in mean performance across cross-validation folds on the x-axis, and the p-value for a t-test comparing the performance distributions on the y-axis, between the “control” feature set and the “drop target” feature set without the target gene, across the 84 genes in the Vogelstein gene set where we could train classifiers for expression and methylation data:*

*Each point in this plot is a gene, and most of the points cluster around the origin, indicating that there is little to no change in performance when the “target gene” is left out of the feature set used to predict mutation status. After multiple testing correction, none of the genes show significant differences in their performance distributions at a testing threshold of 0.05. We can conclude that expression levels of the target gene do not seem to be particularly important to the performance of our gene expression-based classifiers.*

ii. Investigate whether the prediction is driven by the expression landscape of the tissue of origin. To this end, the analysis should be repeated with expression levels from healthy cells of the tissue of origin of the tumor.

*Reply: We agree with the reviewer that it is important to control for the expression landscape of the tissue of origin. Along these lines, we first want to emphasize that our “baseline” model, which we compare against in all of our experiments, is a model where labels are permuted within each cancer type. The model is otherwise trained in the same way, and using the same input features, as the models using the “true” labels. The idea behind this particular baseline, where the proportion of mutated samples in each cancer type is kept constant while the association between labels and sample-specific information is scrambled, is precisely to control for the predictive power of the tissue/cancer type of origin aside from any sample-specific -omics information. We believe that all of the classifiers we identify in our study as* *being “significantly predictive” are an improvement on the cancer type-level predictive ability, by virtue of the way we defined our baseline.*

*It may also be worth mentioning that most of the tumor samples in TCGA (and thus most of the tumor samples that we are looking at in this study) are carcinomas - the expression landscape in normal tissues of origin for carcinomas tend to be very similar to one another and thus would not confer much tissue-specific information.*

*Still, we were curious what the predictions for our classifiers would look like on some of the healthy samples provided in TCGA. The following figure shows predictions for our TP53 mutation classifier trained using RNA-seq data (one of the best-performing classifiers in the present study, and one that has been extensively noted by previous studies) for non-mutated tumor samples, mutated tumor samples, and normal samples, in that order:*

*As expected, we can see that predictions for non-mutated samples tend to be close to 0, and predictions for mutated samples tend to be close to 1 – this is unsurprising since the model was trained on this data. Predictions for the normal samples, however, seem to be spread across the range of probabilities, with a median value around 0.4 and relatively few extreme values. This isn’t terribly surprising since the normal samples are “out of distribution”; that is, the model wasn’t trained on them, and the tumor samples (both mutated and non-mutated) are considerably different from a gene expression perspective. The wide range of predictions for the normal samples suggests that the tissue of origin is probably not driving prediction performance, at least in the case of TP53.*

2. Along similar lines, what are the most important features (i.e. largest regression coefficients)?

*Reply: We fully agree that including the selected features/coefficients from our models would be useful. For the figures in the paper, we evaluate each of our models using 2 replicates of 4-fold cross-validation. For each of these folds (train/test splits), we further split the training set into train and validation sets to select hyperparameters, and evaluate the models on the test set to get the results in the paper. Because we are evaluating performance over multiple folds, it is not perfectly straightforward to get a single set of regression coefficients, since we have a (potentially different) set of coefficients for each cross-validation fold.*

*In order to synthesize these results into a single model for each gene in each data type, we selected one of the 8 sets of hyperparameters (from the 8 best models, 1 per CV fold) at random, with probability proportional to performance (AUPR) on the validation set used to select the hyperparameters, described above (so test set performance is not used here). We then used the selected hyperparameters to train a single model on the entire dataset, and we have made the results available on Figshare and in the supplement of the revised manuscript. The coefficients of the final models trained on gene expression data are available at* [*https://doi.org/10.6084/m9.figshare.19576012*](https://doi.org/10.6084/m9.figshare.19576012)*, and the hyperparameters used to train the final models, i.e. regularization strength and L1 vs. L2 penalty ratio, are available at* [*https://doi.org/10.6084/m9.figshare.19576048*](https://doi.org/10.6084/m9.figshare.19576048) *, for each gene in the “all data types” comparison described in Figure 5. The methods used to generate these models, and the contents of the files, are described in detail in the revised supplement. We believe this will serve as a valuable resource for future work.*

3. p.7, l. 6: The current COSMIC cancer gene census contains 578 tier 1 genes that have been associated with cancer (<https://cancer.sanger.ac.uk/cosmic/census?tier=1>). Why do the authors restrict to the list given in Vogelstein et al. from 2013, which only contains 127 genes and is further reduced to only 85? In particular, the extended list will contain a lot more DNA repair genes.

*Reply: We appreciate the suggestion of the COSMIC Cancer Gene Census as a source of genes having more diverse functions and roles in cancer. As described in our response to Reviewer #1’s second point above, we constructed a larger “merged” set of cancer genes consisting of the union of the genes from Vogelstein et al. 2013, COSMIC CGC, and driver genes identified as “pan-cancer” in Bailey et al. 2018. We were able to predict mutation status accurately in many of these additional genes. In contrast with our results from only Vogelstein et al., we observed that gene expression tended to perform the best of the data types we tested on this larger set of genes.*

4. p. 13, l. 43: Why is it that in this paragraph 100 Vogelstein genes were analyzed instead of 85 or less (p. 7, l.7)?

*Reply: This was a typo; we thank the reviewer for pointing out the inconsistency. This should have been 84 genes, to match the expression/methylation experiments shown in Figure 3. We have updated all of the numbers in the paper to correspond to the results of the “merged” gene set experiments described in the response to Reviewer #1.*

5. p. 7, l. -1: "we included only cancer types with at least 15 mutated samples and at least 5% mutated samples, which we refer to here as "valid" cancer types." Why was there an exclusion criterion for entire cancer types (as opposed to just filtering based on sample properties)?

*Reply: This is a great point; the decision to filter by cancer type rather than just filtering the whole dataset or filtering individual samples is largely a hold-over from previous pan-cancer modeling papers from our group (e.g. Way et al. 2020, reference 13 in the original manuscript, used a similar approach). On the individual sample level, we are currently filtering hypermutated samples from our dataset, since these are likely to be false positives (i.e. mutations in the target gene are not likely to be driving cancer function/progression in these samples), but we are otherwise not filtering out any particular samples from the dataset.*

*One potential alternate approach would be to choose genes to train classifiers for based on properties of the entire dataset, rather than properties of individual cancer types. In other words, we could apply our mutated sample count/proportion thresholds to a particular gene across all cancer types, and train classifiers only for those genes that make the thresholds. We tried this filtering approach with thresholds of 100 total samples mutated and 1% of all samples mutated, which resulted in 182 of the 511 genes in the “merged” cancer gene set that we trained classifiers for (in other words, this is a fairly stringent filter). We then trained our classifiers across all cancer types (“per-gene” filtering), as compared to filtering separately for each cancer type as in the current version of the study (“per-cancer type” filtering). The following table compares performance for the two filtering approaches, across all 6 -omics data types, at a significance cutoff of α=0.001:*

| **Data type** | **Per-cancer type results** | **Per-gene results** |
| --- | --- | --- |
| Gene expression | 79/217 (36.4%) | 81/182 (44.5%) |
| 27k methylation | 31/217 (14.2%) | 16/182 (8.8%) |
| 450k methylation | 42/217 (19.4%) | 1/182 (0.6%) |
| RPPA | 33/217 (15.2%) | 41/182 (22.5%) |
| microRNA | 25/217 (11.5%) | 25/182 (13.7%) |
| mutational signatures | 2/217 (0.9%) | 7/182 (3.9%) |

*The most striking difference is the decrease in performance for both DNA methylation data types for the new (“per-gene”) filtering approach. We have not comprehensively explored the reasons for this, but our intuition is that since DNA methylation patterns are more tissue-specific than gene expression patterns, the addition of “irrelevant” cancer types in the per-gene approach is more detrimental to predictions using DNA methylation than using gene expression and other data types. In general, however, the results are similar in spirit to the current study, with expression performing comparatively well and RPPA performing reasonably well relative to its much smaller dimensionality.*

*Since the relative results between data types are fairly similar, we plan to keep the figures in the main paper using the “per-cancer type” approach we started with, but we have included these results with the “per-gene” approach in Supplementary Figure 11, along with a brief explanation of the difference in filtering methods.*

6. p. 12, l. 7: It is stated that the set of 85 random genes contains eight of the Vogelstein gene set. This implies that the total number of genes that were used (and from which the random genes were drawn) is only on the order of 1,000 genes. What happened with the remaining ~18,000 human protein-coding genes? Why were they excluded?

*Reply: We thank the reviewer for pointing this out, and we agree that this should be clarified. Rather than drawing the “random” gene set from the set of all protein-coding genes, many of which may have somatic mutations in no TCGA samples or very few samples, we restrict to genes with at least one “valid” cancer type, using the same thresholds that we use to include or exclude cancer types for our cancer mutation classifiers (at least 15 mutated samples and at least 5% mutated samples). This results in a set of 1,348 candidate genes, which we sample from uniformly at random.*

*In the revised manuscript, we have added some clarification to the description of how we sampled random genes for this experiment to the Results section (page 11, “We compared performance…”), and pointed readers to the extended description of the sampling process in the Methods section.*

7. Figure 7: How come the models that include more features sometimes perform worse than nested models with fewer features? In particular, gene expression on its own seems to significantly outperform the models that include expression plus additional variables. Is this due to the choice of hyperparameters or due to something else?

*Reply: This is a good question, and something that we agreed was important to explore in more detail. In the current version of the study, we chose fixed hyperparameter ranges and used these ranges for both the single-omics and multi-omics models. In order to rule out the possibility of the hyperparameter ranges being the cause of the relative underperformance of the multi-omics models, we also tried using Bayesian optimization (as implemented in the “scikit-optimize” Python package) to choose hyperparameters, for both the single-omics and multi-omics models. Bayesian optimization methods attempt to find the optimal combination of hyperparameters by constructing a probabilistic model of the hyperparameter space, balancing “exploration” of hyperparameter combinations that haven’t been queried and “exploitation” of hyperparameter settings that have been previously shown to perform well.*

*For each gene and each data type or combination of data types, we ran 80 iterations of Bayesian optimization to select hyperparameters on our validation sets, using starting ranges of alpha = [1 x 10^-6^, 1 x 10^6^] and l1_ratio = [0, 1]. The following figure compares our previous grid search results (blue boxes) with the Bayesian optimization results (orange boxes):*

*We can see here that in general, the parameters chosen using Bayesian optimization tend to outperform the parameters chosen using grid search, suggesting that the search is implemented correctly. If we directly compare the best single-omics model with the best multi-omics model, as in Figure 7A, for the models that used Bayesian optimization, the results are as follows:*

*As we saw before, none of the differences between single- and multi-omics models are statistically significant. So, even with a more comprehensive hyperparameter search method, we saw the same result, suggesting that the hyperparameter ranges we chose are most likely not the reason for the lack of improvement with multi-omics modeling. (Of course we could always run the Bayesian optimization for* *more iterations, but in our 2-dimensional hyperparameter space we assume 80 iterations is likely to find a fairly good solution.)*

8. p. 9, l. 40: Here, it is mentioned that individual cancer types were analyzed as well. The only figure that shows results for individual cancer types is Suppl. Fig. 12. However, this figure is not mentioned in the main text. Also, it only shows five cancer types and only the predictions for survival. The analysis of the other cancer types is missing, and so is the analysis of the prediction of cancer-type-specific driver mutations.

*Reply: Thank you for pointing this out. The original purpose of this figure was an exploratory analysis, to validate the survival prediction/Cox regression implementation outside of the pan-cancer setting (specifically, to make sure that the cancer type covariates/dummy variables weren’t the only factor driving predictive performance). We agree that the figure does not add any significant information to the main text, so we have removed it in the revised manuscript.*

*In general, we agree that an analysis of mutation prediction in specific cancer types would be interesting. However, this would generate a large amount of data (approximately, 33 cancer types in TCGA x 6 data types x 500 cancer genes), and the current study is already quite broad. Since previous work, in our group and others, has shown that a pan-cancer approach tends to work well for the mutation prediction problem, we believe that taking a pan-cancer perspective here is appropriate. In other words, our main focus in this study is to benchmark the relative information content and functional relevance of data types in TCGA, and pan-cancer mutation prediction and survival prediction are sufficient to achieve this in our view.*

9. Suppl. Fig. 12: It is not quite clear to me what is the difference in the prediction of survival by using, say, gene expression in the upper panel vs in the lower panel. Yet, in the upper panel the -omics datasets can predict survival better than the baseline covariates (patient age and mutation count), while in the lower panel they are performing worse. Are these not always single-observable regressions?

*Reply: The primary difference between the upper and lower panels in Figure 12, other than the collection of -omics data that is used, is the set of samples that the experiments are run on. For the upper panels of cancer type-specific results, we are using all samples in TCGA that are profiled for expression, 27K methylation, and 450K methylation, resulting in about 8,000 samples. For the lower panels, we also require that all the samples are profiled for RPPA, microRNA, and mutational signatures data, which restricts us to about 5,000 samples: 3,000 fewer than we had in the upper panels. This difference in the sample set is presumably what changes the results relative to the baseline, since the features used to predict survival are indeed the same.*

*Generally, in our study, this difference in the collection of samples we are restricted to is the reason why we separate the expression/methylation experiments (e.g. Figure 3) from the “all data types” experiments (e.g. Figure 5) rather than just comparing all data types from the beginning. The overall consistency in the results for the expression and methylation data types between figures 3 and 5 suggests that our conclusions shown in the main figures/data are robust across the different sets/numbers of samples, which we believe is an important result, but on the individual cancer type level shown in Supp. Fig 12 this is not necessarily the case.*

10. I think the most interesting application of the approach would be to predict mutation status of all ~19,000 human protein-coding genes to see if the association with expression level can identify novel cancer driver genes. But it is appreciated that the multiple testing burden would probably overwhelm any signal (including the one on the known cancer driver genes).

*Reply: We agree with the reviewer that predictions for all protein-coding genes would be a useful resource. However, based on the results of our experiments with the larger set of cancer-related genes (as described in several of the points above), we have seen that in increasing the size of our cancer gene set approximately 3-fold, there is a considerable drop in the ratio of genes that pass our significance filters to those that do not. For example, for gene expression, we saw that 40/84 genes (47.6%) passed the threshold for the Vogelstein et al. gene set, and 113/272 genes (41.5%) passed the threshold for the merged cancer gene set. Some of this may be attributable to biological factors (for instance, the Vogelstein genes may contain more well-established/high effect size drivers, and the added genes from COSMIC CGC/Bailey et al. could have smaller or more cancer type-specific effects making their mutation status harder to predict), but it is likely that this decrease in overall performance also stems in part from the increased multiple testing burden, as the reviewer pointed out.*

*In our opinion, choosing a fairly large/permissive set of genes with established cancer relevance, as we have done in the revised version of our study, is the best way to navigate the tradeoff between missing less well-studied genes that may contain novel or interesting signal and overwhelming signal due to a high multiple testing burden.*

11. p. 18, l. 5: 36 --> 34

12. p. 18, l. 12: There are no red boxes in Figure 6.

*Reply: Thank you for pointing these out - both of these mistakes are fixed in the revised paper.*

**Second round of review**

**Reviewer 1**

Authors have addressed all my concerns in the revised manuscript.

**Reviewer 2**

1.ii. I agree with the authors that gene expression of the tissue of origin was already accounted for by randomizing at the cancer-type level. I still enjoyed the results from the test on TP53 that they produced.

2. The authors have provided the regression coefficients for the models trained on gene expression data (and the hyperparameters for the "all data types" comparison). Can they also provide the coefficients for training on methylation data and the "all data types" scenario? This can be done by adding them to the same website that contains the others.

I think the paper has gained from including the larger cancer driver gene set and thank the authors for addressing all my concerns. Overall, I think it provides an interesting study of the interaction between expression or methylation patterns and cancer driver genes.

**Authors Response**

**Point-by-point responses to the reviewers’ comments:**

Reviewer #2:

1.ii. I agree with the authors that gene expression of the tissue of origin was already accounted for by randomizing at the cancer-type level. I still enjoyed the results from the test on TP53 that they produced.

2. The authors have provided the regression coefficients for the models trained on gene expression data (and the hyperparameters for the "all data types" comparison). Can they also provide the coefficients for training on methylation data and the "all data types" scenario? This can be done by adding them to the same website that contains the others.

I think the paper has gained from including the larger cancer driver gene set and thank the authors for addressing all my concerns. Overall, I think it provides an interesting study of the interaction between expression or methylation patterns and cancer driver genes.

*Reply: We believe that we were able to address all of the issues raised in the previous revision. In particular, we have included the coefficient and hyperparameter information for the remaining data types besides gene expression (including the two methylation data types, and all other data types), referenced in the "Availability of data and materials" section of the most recent manuscript version and described in more detail in the supplement.*
